# Increased HOX C13 expression in metastatic melanoma progression

**DOI:** 10.1186/1479-5876-10-91

**Published:** 2012-05-14

**Authors:** Monica Cantile, Giosuè Scognamiglio, Annamaria Anniciello, Marisa Farina, Giusy Gentilcore, Clemente Santonastaso, Franco Fulciniti, Clemente Cillo, Renato Franco, Paolo A Ascierto, Gerardo Botti

**Affiliations:** 1 Pathology Unit, Istituto Nazionale Tumori Fondazione “G. Pascale”, via Mariano Semmola, 80131, Naples, Italy; 2 Department of Melanoma, Unit of Medical Oncology and Innovative Therapies, Istituto Nazionale Tumori Fondazione “G. Pascale”, via Mariano Semmola, 80131, Naples, Italy; 3 Department of Experimental Medicine, University of Naples Federico II, via S. Pansini, 80131, Naples, Italy

**Keywords:** HOX genes, Metastases, Melanoma cells

## Abstract

**Background:**

The process of malignant transformation, progression and metastasis of melanoma is not completely understood. Recently, the microarray technology has been used to survey transcriptional differences that might provide insight into the metastatic process, but the validation of changing gene expression during metastatic transition period is poorly investigated. A large body of literature has been produced on the role of the HOX genes network in tumour evolution, suggesting the involvement of HOX genes in several types of human cancers. Deregulated paralogous group 13 HOX genes expression has been detected in melanoma, cervical cancer and odonthogenic tumors. Among these, *Hox C13* is also involved in the expression control of the human keratin genes *hHa5* and *hHa2*, and recently it was identified as a member of human DNA replication complexes.

**Methods:**

In this study, to investigate *HOX C13* expression in melanoma progression, we have compared its expression pattern between naevi, primary melanoma and metastasis. In addition *HOXC13* profile pattern of expression has been evaluated in melanoma cell lines.

**Results:**

Our results show the strong and progressive *HOX C13* overexpression in metastatic melanoma tissues and cytological samples compared to nevi and primary melanoma tissues and cells.

**Conclusions:**

The data presentated in the paper suggest a possible role of *HOX C13* in metastatic melanoma switch.

## Background

Melanoma is the most frequent skin malignancy, with poor prognosis because associated to high metastatic capacity [1]. Currently, although several molecular pathways have been described for melanoma progression, the molecular mechanisms that lead to metastasis development have not completely understood [1]. HOX genes regulate normal embryonic development, cell differentiation and other critical processes in eukaryotic cell life. Several studies have shown that HOX genes play a role in regulating the homeostasis and both proliferation and differentiation of skin cells during development [2-4]. Moreover, recently, it has been better investigated their role in neoplastic transformation in several human tissues [5-10]. In particular the genes belonging to HOX paralogous group 13 seem to have a relevant role in both neoplasia development and progression. Thus *HOX B13* gene deregulation is strongly associated to breast and ovarian carcinogenesis [11-14] and its expression appears cytoplasmic during epidermis embryonic development, and nuclear in differentiated adult epidermal cells, while in epidermal proliferative disorders cytoplasmic expression has been documented [15]. Moreover, we have identified a significant prognostic role of *HOX D13* in pancreatic cancer [16] and recently, it was shown that *HOX A13* plays a relevant role in human hepatocarcinoma progression [17]. Finally, the amplification of 12q13-15, containing *HOX C13* gene and the entire HOX C locus, was associated with a large number of cancers such as sarcomas, glioblastomas, lung, bladder and transitional carcinomas and melanomas [18-25].

In skin homeostatsis *HOX C13* plays a fundamental role, particularly in epithelial component, being mainly involved in the control of nails, hair and hair follicles development. In addition it regulates the transcription of cytokeratins genes tying a highly conserved motif in the promoter of these genes [26-28].

In this study, to investigate *HOX C13* role in melanoma progression, we have compared its expression pattern between juctional, compound and dermic naevi and primary and metastatic human melanoma. Moreover we have evaluated *HOXC13* expression in melanoma cell lines. In particular we have built a melanoma progression Tissue Micro Array (TMA) in order to perform immunohistochemistry analysis of HOX C13 expression. Moreover we have analyzed through quantitative Real Time PCR (qRT-PCR) analysis *HOX C13* mRNA expression in the same series of samples and in melanoma cell lines.

## Methods

### Patients

Histological blocks of cases have been selected in the files of Pathology Unit of National Cancer Institute Fondazione G. Pascale of Naples and all patients were caucasians, and all gave their written informed consent according to the institutional regulations.

This study was approved by the ethics committee of National Cancer Institute “G. Pascale”. Our series included nevi, primary melanomas and corresponding metastatic tissues from the same patients and an independent series of cytologic nodal melanoma metastases. All histological tissue cases were reviewed by three pathologists (AA, RF, GB) according to AJCC classification criteria, using standard tissue sections and appropriate immunohistochemical slides. Moreover medical records have been reviewed for clinical information. Synthethically clinicopathologic parameters re-evaluated in our study for each tumor included patient age at initial diagnosis; tumor size; histologic subtype; sentinel lymphnode status; non sentinel lymphnode metastasis; number of positive lymph nodes; tumor stage; tumor recurrence or distant metastasis; type of surgery.

### Cell lines

The human MEL-Juso lines was cultured in RPMI 1640 medium supplemented with 5.1 ml of L-glutamine and 10% fetal calf serum; WM115 was kept in half Emem supplemented with 2 mM L-glutamine, 1% nonessential amino acids, 1% sodium pyruvate (NaP) and 10% fetal calf serum; SK-MEL, A-375, MZ2, Me-15 and Me-51, and Me-67 were grown in DMEM Ham’s F12 supplemented with 2 mM L-glutamine and 10% fetal calf serum; The HBL-8 and NA-8 were cultured in RPMI 1640 supplemented with 1 mM NaP, 2 mM non-essential amino acids, 2 mM L-glutamine and 10% fetal calf serum. The cells were maintained in an incubator at 37°C in a humidified atmosphere with 5% CO2.

### TMA building

Tissue Micro-Array was built using the most representative areas from each single case of metastasis. All tumours and controls were reviewed by three experienced pathologists (AA,RF,GB). Discrepancies between two pathologists from the same case were resolved in a joint analysis of the cases. Tissue cylinders with a diameter of 0.6 mm were punched from morphologically representative tissue areas of each ‘donor’ tissue block and brought into one recipient paraffin block (3 × 2.5 cm) using a semiautomated tissue arrayer (Galileo TMA).

### HOX C13 Immunostaining

Human hair follicles histological sample were used as positive control. Immunohistochemical and immunocytochemical staining was done on slides from formalin-fixed, paraffin embedded tissues, and cytological samples on thin prep to evaluate the expression of HOX C13 marker. Paraffin slides were then deparaffinized in xylene and rehydrated through graded alchols. Antigen retrieval was performed by microwave pretreatment in 0.01 m citrate buffer for 10 min. After protein block (BSA 5% in PBS 1x), the slides were incubated with primary antibody to human HOX C13 (dilution 1:1200, cod.ab55251, Abcam,Cambridge,UK) over night. Sections were incubated with mouse anti-rabbit or goat anti-mouse secondary IgG biotinylated secondary antibody for 30 min. Immunoreactivity was visualised by means of avidin–biotin–peroxydase complex kit reagents (Novocastra, Newcastle, UK) as the chromogenic substrate. Finally, sections were weakly counterstained with haematoxylin and mounted. Human hair follicles were used as positive controls. Irrelevant rabbit or mouse IgG antibodies were applied to negative control. Standard sections for primitive melanoma and melanoma metastasis TMA have been used. Results are interpreted using a light microscope.

### Double staining IHC

For double staining of HMB-45 and HOX C13, deparaffinized sections were microwaved in 1 mmol/L EDTA (pH 8.0) for two cycles of 5 minutes each to unmask epitopes. After treatment with 1% hydrogen peroxidase for 30 minutes to block endogenous peroxide, the sections were subsequently incubated with HOXC13 monoclonal antibody over night at 7°C. Followed by a wash step to remove any excess antibody. The antibody was visualized using the peroxidase detection system consists of EnVision™ FLEX+, Mouse (LINKER) (Dako, SM804) and after wash step EnVision™ FLEX/HRP (Dako, SM802) both for 30 minutes a room temperature. Finally, was used the diaminobenzidine (DAB) as contrasting chromogen.

Antibody denaturation (DNS001H, 1 part A with 3 part B, Biocare Medical, USA) was then performed for 5 min to ensure that the first primary antibody was completely inactivated before applying the second primary antibody. Thus, HMB45 monoclonal antibody (pre-diluited, cod. 790–4366, Ventana Medical System, Tucson, USA) was subsequently applied as a second primary antibody and was incubated for 45 min. HMB45 was visualized using the MACH 2 mouse Ap-polymer (MALP521H, Biocare Medical, USA) for 30 min and incubated with Vulcan fast red for 15 min (FR805H, Biocare Medical, USA).

### Evaluation of immunohistochemistry

For HOX C13 expression on the positive control, we considered nuclear positivity. For HOX C13 on melanoma tissues, cytoplasmatic and nuclear staining have been considered. Melanoma tissues were scored semi-quantitatively by evaluating the proportion of positive tumour cells over the total number of tumour cells (percentage of positive tumour cells per tissue microarray punch). Negative (Score 0), low expression cases, and, high expression cases were recorded when neoplastic cells expressing HOX C13 were between 0 and 10% (Score 1+), lower than 30% (score 2+) and higher than 30% (Score 3+), respectively. For the evaluation of HOX C13 expression in cytologic specimens, we considered the percentage of positive cells by the same score used for assessment of histological samples. For the evaluation of IHC double staining, we considered nuclear HOX C13 (brown) expression and cytoplasmatic HMB-45 (red) expression.

### RNA extraction from fresh cellular sospension and paraffin embedded tissues

The sections obtained from paraffin-embedded samples were incubated at 37°C in the presence of xylene for about 20 minutes. Total RNA was purified using High Pure FFPE RNA Micro Kit (Roche) following the manufacturer’s instructions. Total RNA was isolated from cytological samples and cell lines sospensions, using RNeasy Mini Kit (Qiagen GmbH, Hilden, Germany) following the manufacturer’s instructions. All samples were treated with RNase-free DNase (Qiagen GmbH, Hilden, Germany) to prevent amplification of genomic DNA. A total of 1 μg RNA was subjected to cDNA synthesis for 1 hr at 37°C using the Ready To Go You-Primer First-Strand Beads kit (Amersham Biosciences Europe Gmbh, Freiburg, Germany, cod. 27-9264-01) in a reactionmixture containing 0.5 μg random hexamers (GeneAmp RNA PCR Random Hexamers Set N808-0127 Applied Biosystems, Foster City, CA).

### Real-time PCR

qRT-PCR was performed in a LightCycler system (Roche Molecular Biochemicals, Mannheim, Germany) using TaqMan® analysis. In this system, all reactions were run in glass capillaries with The LightCycler TaqMan Master Mix (cod. 04735536001, Roche Molecular Biochemicals), 10 μl, in a volume of 20 μl containing 2 μl of cDNA and 1 μl of specific TaqMan Gene Expression Assays for human *HOX C13* (RealTime Designer Assay cod. 04162498001, Roche Molecular Biochemicals), according to the manufacturer’s directions. All reactions were performed in triplicate. The thermal cycling conditions included a step of 20 sec at 95° C followed by a 40 cycles of 95° C for 1 sec and 60°C for 20 sec. The comparative C_t_ method was employed to determine the human *HOX C13* gene variation, using as reference gene TaqMan Endogenous Controls Human *ACTB (β-actin*) Endogenous Control (RealTime Designer Assay cod.05532957001, Roche Molecular Biochemicals). We identified a calibrator cell line that represents the unitary amount of the target of interest, consequently the samples express n-fold mRNA relative to the calibrator. Final amounts of target were determined as follows: target amount = 2-C_t_, where C_t_ = [C_t_ (*HOXC13*) − C_t_ (*ACTB*)]_sample_ − [C_t_ (*HOXC13*) − C_t_ (*ACTB*)]_calibrator_.

### Statistical analysis

The association between HOX C13 with other clinico-pathological parameters was conducted using the κ ^2^ and T Student test. The Pearson κ ^2^ test was used to determine whether a relationship exists between the variables included in the study. The level of significance was defined as *P* < 0.05. Overall Survival (OS) and Progression-Free Survival (PFS) curves were calculated using the Kaplan-Meier method. Statistical significance of associations between individual variables and OS and FFS was determined using the log-rank test. All the statistical analyses were carried out using the Statistical Package for Social Science 8.0 software (SPSS Inc., Chicago, IL, USA). OS was defined as the time from diagnosis (first biopsy) to death by any cause or until the most recent follow-up. PFS was measured as the time from diagnosis to the occurrence of progression, relapse after complete remission, or death from any cause.

## Results

### Clinicopathological characteristics of melanoma patients and tumors

Our series of histological samples have been constituted by 48 primary melanoma and corresponding metastases, and 10 nevi (Table 1). Female patients represented from 26 out of 48 (54.2%); Clark level was I in 4% (2/48), II in 33% (16/48) III in 52% (25/48) and IV in 10% (5/48) of cases; Pt was I in 12% (6/48), II in 33% (16/48), III in 14% (7/48), IV in 39% (19/48) of cases; Mitotic rate was 0 in 28% (12/42) and >1 in 71% (30/42) of cases; TILs was absent in 35% (17/48), Non-brisk in 43% (21/48) and Brisk in 20% (10/48) of cases; ulceration was present in 35% (17/48) and absent in 64% (31/48) of cases; Cell Type predominant was epithelioid cells (64%) and Nodular melanoma type was prevalent (50%).

**Table 1:** Clinicopathological characteristics of melanoma patients and tumors and relation with HOXC13 expression

**HOX C13 Score**	**0**	**1**	**2**	**3**	**Sample N°**	
Sex N°						
Male	1 (4,5%)	13 (59,1%)	7 (31,8%)	1 (4,5%)	48	0,945
Female	2 (7,7%)	16 (61,5%)	8 (30,8%)	0 (0%)		
Clark level						
I	0 (0%)	2 (100,0%)	0 (0%)	0 (0%)		
II	0 (0%)	10 (62,5%)	6 (37,5%)	0 (0%)	48	0,1111
III	3 (12,0%)	13 (52,0%)	9 (36,0%)	0 (0%)		
IV	0 (0%)	4 (80,0%)	0 (0%)	1 (20,0%)		
Pt						
I	0 (0%)	6 (100,0%)	0%	0%		
II	0 (0%)	6 (37,5%)	9 (56,3%)	1 (6,3%)	48	0,0001
III	3 (42,9%)	2 (28,6%)	2 (28,6%)	0 (0%)		
IV	0 (0%)	15 (78,9%)	4 (21,1%)	0 (0%)		
Mitotic rate per mm2						
0	0 (0%)	9 (75,0%)	2 (16,7%)	1 (8,3%)		
>1	3 (10,0%)	16 (53,3%)	11(36,7%)	0 (0%)	42	0,133
TILs						
Absent	1 (5,9%)	10 (58,8%)	5 (29,4%)	1 (5,9%)		
Nonbrisk	0 (0%)	13 (61,9%)	8 (38,1%)	0 (0%)	48	0,339
Brisk	2 (20,0%)	6 (60,0%)	2 (20,0%)	0 (0%)		
Ulceration						
Present	1 (5,9%)	11 (64,7%)	5 (29,4%)	0 (0%)	48	1
Absent	2 (6,5%)	18 (58,1%)	10(32,3%)	1 (3,2%)		
Predominant cell type						
Epithelioid	1 (3,2%)	22 (71,0%)	8 (25,8%)	0 (0%)		
Spindle	2 (20,0%)	2 (20,0%)	5 (50,0%)	1 (10,0%)	48	0,045
Other	0 (0%)	5 (71,4%)	2 (28,6%)	0 (0%)		
Melanoma Type						
Superficial spreading melanoma	0 (0%)	7 (77,8%)	2 (22,2%)	0 (0%)		
Nodular melanoma	3 (12,5%)	12 (50,0%)	9 (37,5%)	0 (0%)	48	0,286
Acrolentiginous melanoma	0 (0%)	6 (75,0%)	1 (12,5%)	1 (12,5%)		
Lentigo maligna melanoma	0 (0%)	4 (57,1%)	3 (42,9%)	0 (0%)		
Sentinel lymph node						
negative	3 (11,1%)	16 (59,3%)	7 (25,9%)	1 (3,7%)		
micrometastases	0 (0%)	4 (80,0%)	1 (20,0%)	0 (0%)	48	0,431
subcapsular	0 (0%)	7 (77,8%)	2 (22,2%)	0 (0%)		
diffuse	0 (0%)	2 (28,6%)	5 (71,4%)	0 (0%)		
Nevus	8(80,0%)	2(20,0%)	0(0%)	0(0%)	10	
Primary melanoma	3(6,3%)	29(60,4%)	15(31,3%)	1(2,1%)	48	
Metastasis						
1	2 (33,3%)	2 (33,3%)	2 (33,3%)	0 (0%)		
2	0 (0%)	10 (83,3%)	1 (8,3%)	1 (8,3%)	48	0,013
3	1 (3,3%)	17 (56,7%)	12(40,0%)	0 (0%)		

In addition, 20 cytological lymph node metastases of melanoma samples were selected for our study and clinical characteristics of patients with respect to HOX C13 expression are shown in Table 2.

**Table 2:** Clinical characteristics of cytological melanoma samples and relation with HOXC13 expression

**HOX C13 Score**	**0**	**1**	**2**	**3**	**Sample N°**
Sex N°					
Male	1 (9,1%)	1 (9,1%)	5 (45,5%)	4 (36,4%)	20
Female	0 (0%)	2 (22,2%)	2 (22,2%)	5 (55,6%)	
Age					
<60	0 (0%)	2 (22,2%)	3 (33,3%)	4 (44,4%)	20
>60	1 (9,1%)	1 (9,1%)	4 (36,4%)	5 (45,5%)	
pT					
I	0 (0%)	0 (0%)	1(100%)	0(0%)	20
II	0 (0%)	0 (0%)	0 (0%)	5 (100%)	
III	1 (14,3%)	1 (14,3%)	3 (42,9%)	2 (28,6%)	
IV	0 (0%)	2 (28,6%)	3 (42,9%)	2 (28,6%)	
Lymph node localization					
axillary	1 (11,1%)	0 (0%)	4 (44,4%)	4 (44,4%)	20
inguinal	0 (0%)	2 (28,6%)	2 (28,6%)	3 (42,9%)	
iliac region	0 (0%)	1 (100%)	0 (0%)	0 (0%)	
scapulary	0 (0%)	0 (0%)	0 (0%)	1 (100%)	
supraclavicular	0 (0%)	0 (0%)	1 (50%)	1 (50%)	

### Expression of HOXC13 protein in primary and metastatic melanoma tissues and cytological samples

**Figure 1:**
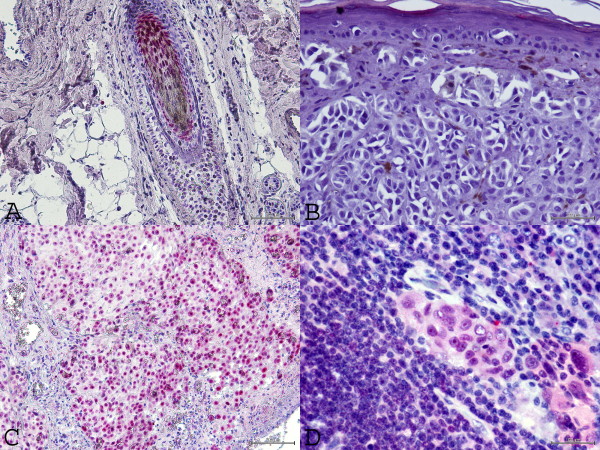
**Localization of HOX C13 protein in human hair follicles: immunopositivity (red) in cortex (co), in cuticle (cu), in hair papilla (hp), and in germinative cells (gc).** Immunonegativity in medulla (me), in outer root sheath (ors), in connective sheath (cs) and inner root sheath (irs).

**Figure 2:**
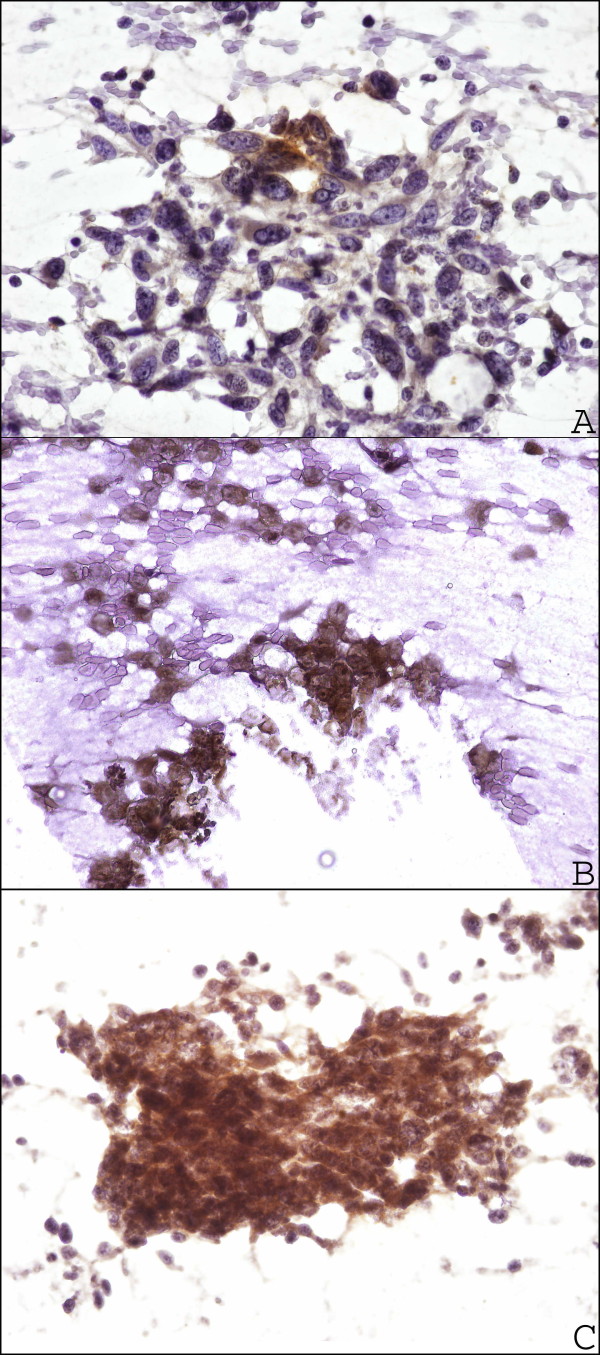
**HOX C13 immunostaining in melanoma tissues. A:** HOX C13 immunopositivity in hair bulb follicle as positive control (20X); **B:** HOX C13 immunonegativity in primary melanoma sample (40X); **C:** High HOX C13 immunopositivity in melanoma metastasis sample score 3+ (20X); **D:** High HOX C13 immunopositivity in melanoma micro-metastasis sample score 3+ (60X).

**Figure 3:**
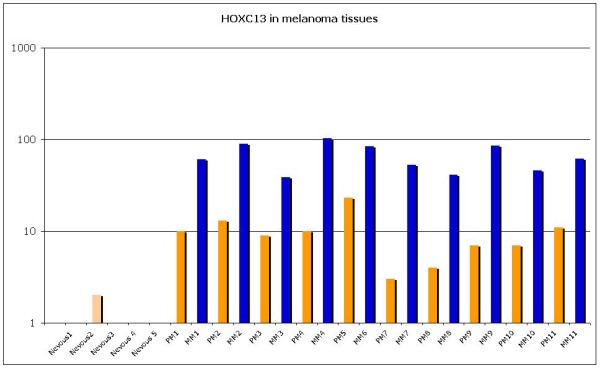
**HOX C13 immunostaining in limphnode melanoma cytological samples. A:** HOX C13 immunopositivity score 1+ (60X); **B:** HOX C13 immunopositivity score 2+ (60X); **C:** HOX C13 immunopositivity score 3+ (60X).

**Figure 4:**
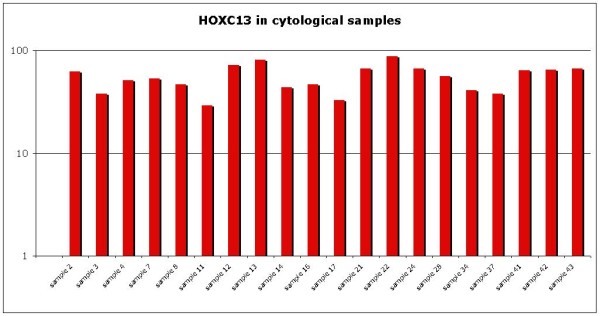
**HOX C13/HMB-45 double staining IHC in primary melanoma tissues. A:** HOX C13 immunonegativity in HMB-45 positive (red) primary melanoma (10X); **B:** details of melanoma cells in papillary dermis (20X); **C:** details of melanoma cells in reticular dermis (20x).

**Figure 5:**
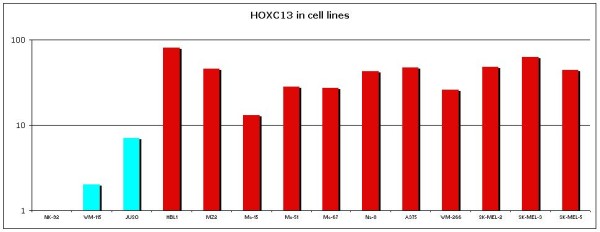
**HOX C13/HMB-45 double staining IHC in metastatic melanoma tissues. A:** Cytoplasm HMB-45 immunopositivity (red) and nuclear HOX C13 immunopositivity (brown) in nodal metastasis (20X); **B:** Cytoplasm HMB-45 immunopositivity (red) and nuclear HOX C13 immunopositivity (brown) in nodal subcapsular micrometastasis (40X) **C:** Cytoplasm HMB-45 immunopositivity (red) and nuclear HOX C13 immunopositivity (brown) in dermal metastasis (40X).

For HOX C13 expression on the positive control, we detected nuclear positivity (red) in cortex, cuticle, dermal papilla and germinative cell compartment of hair follicle (Figure 1). In all dermic naevus the expression was absent, while it was low/moderate (5/15%) in most primary melanoma samples. Moreover, in primary melanomas the expression of HOX C13 was negative in three cases (6.3%), in 29 cases score 1 positivity (60.4%), in 15 cases (31.5%) scores 2 positivity and score 3 (2.1%) in one case have been observed. Cytoplasmic positivity gnerally has been observed with focal nuclear expression. In corresponding metastatic tissues specimens, in 29 cases HOX C13 expression appears with score 3 (60.4%), in 11 cases (22.9%) with score 2, and only in 6 cases with score 1. In metastatic cells a significant higher nuclear positivity has been observed (Figure 2, Table 1). Table 3 presents data on the expression of HOX C13 in metastases of different bodily localization, where it is shown the predominant expression (score 3+) in lymph node metastases (73.3%). Furthermore, HOX C13 expression was very high (score 3+) in most cytological samples of nodal melanoma metastasis and with prevalent cytoplasmatic localization (80%) (Figure 3, Table 3). In addition, we developed a double staining with melanoma-specific marker HMB-45 in primary and metastatic melanoma samples (Figures 4 and 5) and found that all HOX C13-positive tumour cells also expressed HMB-45.

**Table 3:** Relation between HOX C13 expression and metastases localization

**HOX C13 Score**	**0**	**1**	**2**	**3**	**Sample N°**
Lymph node met.	0 (0%)	2 (13,3%)	2 (13,3%)	11(73,3%)	15
Skin met.	1 (8,3%)	0 (0%)	4 (33,3%)	7 (58,3%)	12
Visceral met.	1 (4,8%)	4 (19,0%)	5 (23,8%)	11(52,4%)	21
					48

### HOXC13 mRNA quantification in histological and cytological melanoma samples and in primary and metastatic cell lines

**Figure 6:**
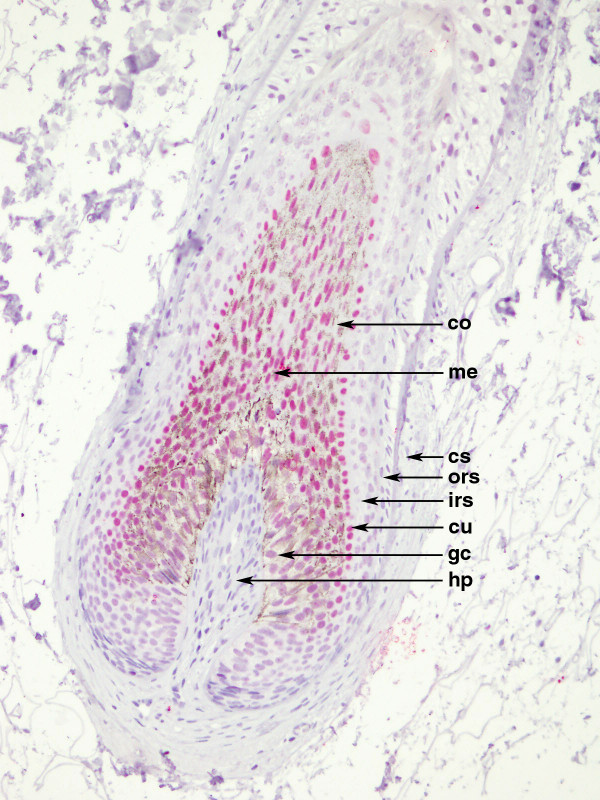
**HOX C13 Real Time expression in primary and metastatic melanoma tissues.** All reactions were performed in triplicate and data are expressed as mean of relative amount of mRNAs levels.

**Figure 7:**
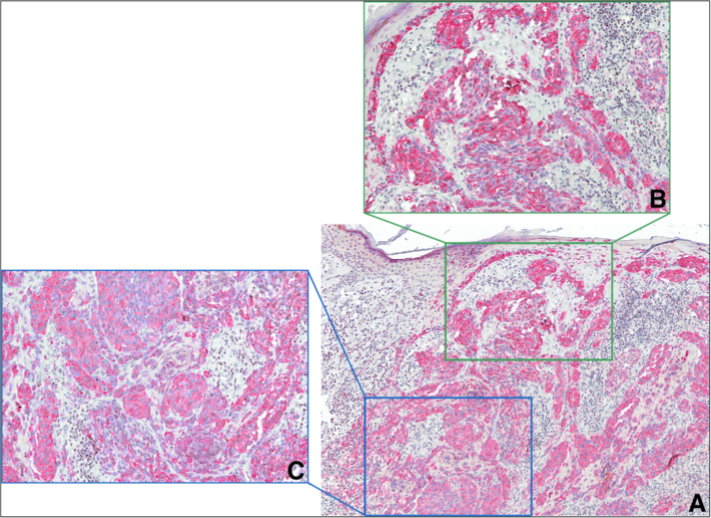
***HOX C13*Real Time expression in cytological melanoma samples.** All reactions were performed in triplicate and data are expressed as mean of relative amount of mRNAs levels.

**Figure 8:**
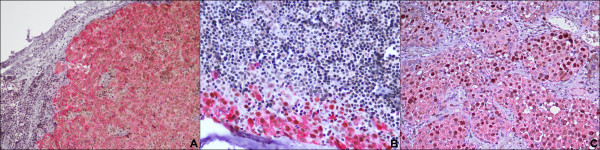
***HOX C13*Real Time expression in primary and metastatic cell lines.** All reactions were performed in triplicate and data are expressed as mean of relative amount of mRNAs levels.

*HOX C13* gene expression was evaluated on selected primary and metastatic melanoma tissues by qRT-PCR. In nevous samples *HOX C13* mRNA expression appears silent, except in one case (Figure 6). In primary samples it was observed a low expression in samples 7 and 8, while it was moderate in remainder melanoma tissues analyzed (between five and ten-fold increase). In all corresponding metastatic melanoma samples it was observed a significant increase in *HOX C13* mRNA expression (between ten and hundred-fold increase) (Figure 6). Gene expression analysis was also performed on the same cytological samples used for immunostaining, and *HOX C13* appears over-expressed in all specimens (between ten and hundred-fold increase) (Figure 7). RT-PCR quantification was also performed on selected commercially primary and metastatic melanoma cell lines. In WM-115 and JUSO primary melanoma cultures *HOX C13* mRNA expression appears moderate (between three and ten-fold increase), while in all metastatic cell lines it was observed a significant increase of gene expression (between ten and hundred-fold increase) (Figure 8).

### Statistical analysis

χ square analysis has shown a significant association between absent/low HOXC13 expression and pT1-2 stage group of patients (*p* = 0.0001) and epithelioid phenotype (*p* = 0.045) (Table 1). HOX C13 expression in metastasis were significant higher in metatasis with respect to primitive melanomas (*p* = 0.013) (Table 1). In cytological samples has shown a prevalent score 3+ HOX C13 expression, independently of the clinical characteristics of patients (Table 2). Survival analysis of melanona cases included in TMA related to the HOX C13 expression did not reveal statistically significant results.

## Discussion

Metastasis development is the prognostic key of melanoma progression, but the molecular mechanisms that determine the ability of neoplastic cells to separate from the primary tumors, conquer the bloodstream, and invade new tissues are still poorly known. The entire network of HOX genes plays a crucial role in embryonic development, and is involved in controlling of cell identity phenotype and three-dimensional organs and tissues formation [28-34]. Alterations of specific groups of HOX genes can be associated with neoplastic transformation of different tissues and organs, such as breast, kidney, colon, lung, thyroid, bladder, prostate, lymphoid organ and skin tissue [6]. Thus the possibility of identifing the levels of expression of these genes may be important for diagnosis and prognosis of these pathologies. HOX genes belonging to paralogous group 13 are able to generate mutations not compatible with life, as in the case of synpolydactyly [35], and hand-foot-genital syndrome [36]. In addition they are directly associated with the processes of cell proliferation and differentiation in vivo and vitro and cancer progression [11,37]. In this study we investigated the role played by *HOX C13*, located a chromosomal area, 12q13-15, widely studied in human tumorigenesis.

Our results show the strong and progressive over-expression of HOX C13 in melanoma metastatis when compared to nevi and primary melanomas, suggesting the HOX C13 role in metastatic melanoma switch. These results have een confirmed in melanoma metastasis cell lines. There are few indications in the literature regarding the role of HOX genes network in melanoma development and in the progression of this neoplasia.

The first work carried out in vitro on metastatic melanoma cell lines showed the aberrant expression of a block of genes located at 5 end of the HOX C locus (*HOX C10, C11*, and *C13*) directly related to alteration of their adhesive properties (*integrins* and *ICAM-I*), to a Gln-Arg mutation in the codon 61 of the *N-RAS* gene, and to the expression of *IL-1a, IL-6*, and *TNF-alpha*[38]. After, Maeda et al. .have been shown the alteration of specific HOX genes in melanoma genesis and metastasis [39].

Furthermore, more recently a gene expression profiling of primary, non-metastatic cutaneous tumors and metastatic melanoma has resulted in the identification of several genes that may be centrally involved in the progression and metastatic potential of melanoma identyfing several HOX genes as fundamental in this process [40]. To conduct our analysis we selected a range of human biological samples representative of malignant melanoma progression. The data obtained showed that HOX C13 expression, is significantly increased in metastatis analyzed compared to the corresponding primary tumors of the same patients.

Our data conflicted with previously published data [39] that showed higher expression of *HOXC13* in nevi and pT1/pT2 as compared to pT4 and decreased expression in more advanced tumors. However, in the paper of Maeda et al. were not used metastatic samples and corresponding primary tumors from the same patients, representing progression of the disease. Moreover, all our data have been produced for immunohistochemistry and confirmed by qRT-PCR, and this has allowed us to verify that alterations in *HOX C13* expression are maintened both gene and protein level. In fact, often the correspondence between the two expression profiles are not comparable.

## Conclusions

The search for new molecular markers that allow to select the tumor cell clones with respect to their aggressiveness and their ability to metastasize, is certainly one of the most important goals in melanoma biomedical research, and in this context, the HOX genes represent ideal targets for such studies. In fact, *HOX C13* could be an ideal marker on which to interfere at both mRNA and protein level.

We speculate that our data may represent an important element for the development of potential therapies targeted against the activities of *HOX C13*. In fact, recently, it was shown that several molecules, as antisense oligonucleotide (ASO) and small interfering RNA (siRNA), could be able to interact with crucial genes in the modifying tumoral cells growth and proliferation and thus representing ideal tools for targeted therapy [41].

Moreover, Pre-B-Cell-Leukemia transcription factors (*PBX*), a family of genes belonging to the TALE superfamily of homeobox genes, [42,43] are able to interact with a subset of HOX proteins, influencing the regulation of transcription and are required for many aspect of the Hox function. The possibility of targeting the post-translational interaction between HOX proteins and their PBX cofactors, through the short peptide HXR9 that is able to bind a conserved six-amino acid sequence in the HOX proteins, to block in vivo and vitro the HOX/PBX-regulated gene expression of melanoma cells has been shown recently [44].

In conclusion, targeting HOX/PBX interaction may represents a potential alternative or addition to current cancer therapies when drug resistance develops also in therapies against melanoma progression.

## Competing interest

All authors of this manuscripts report no conflict of interest with respect to any financial or personal relationships with other people or organizations that could inappropriately influence our work.

## Authors’ contributions

PAA RF and CC were responsible for the conception and design of the study. MC, GS, AA, MF, GG, FF, CC, and GB were responsible for provision of study materials or patients. GS, MF, collected and assembled data and samples for the Tissue MicroArray and immunohistochemical analysis. MC, MF, GS, were responsible for the Real Time PCR analysis. GB and RF were involved in statistical analysis. AA, FF, RF and GB were responsible for immunohistochemical evalutation. All authors were involved in manuscript writing and provided final approval of the manuscript.

## Role of the funding source

This study has no funding source and no other involvement.
